# Emergency upper gastrointestinal endoscopy performed safely in a patient with COVID‐19 with suspected hemorrhagic shock

**DOI:** 10.1002/deo2.2

**Published:** 2021-04-28

**Authors:** Yoshitsugu Misumi, Yuki Nitta, Kouichi Nonaka, Masatoshi Kawana, Ken Arimura, Katsutoshi Tokushige, Kazunari Tanabe

**Affiliations:** ^1^ Department of Digestive Endoscopy Tokyo Women's Medical University Tokyo Japan; ^2^ Department of Gastroenterology Tokyo Women's Medical University Tokyo Japan; ^3^ Department of General Medicine Tokyo Women's Medical University Tokyo Japan; ^4^ Department of Respiratory Medicine Tokyo Women's Medical University Tokyo Japan; ^5^ Department of Urology Tokyo Women's Medical University Tokyo Japan

**Keywords:** COVID‐19, emergency endoscopy, gastrointestinal endoscopy, infection control, personal protective equipment

## Abstract

Coronavirus disease 2019 caused by severe acute respiratory syndrome coronavirus 2 has spread explosively throughout the world and has since been declared a pandemic by the World Health Organization. Although it is recommended that upper gastrointestinal endoscopies either be postponed or canceled during the pandemic because of their high risk of aerosol generation, this does not apply in emergency cases, which may include patients with coronavirus disease. In this case report, we describe the safe undertaking of an emergency upper gastrointestinal endoscopy in a patient with suspected hemorrhagic shock who tested positive for the severe acute respiratory syndrome coronavirus 2 using the polymerase chain reaction. We performed the procedure in the contamination zone of a specialized coronavirus disease ward with prespecified zones. Full personal protective equipment was worn during the procedure, as recommended by various academic societies, and careful attention was paid to the sterilization of all equipment after the procedure. Thus, emergency endoscopies can be performed safely in patients with coronavirus disease in a suitable environment by using appropriate personal protective equipment and by handling the equipment appropriately.

## INTRODUCTION

Coronavirus disease 2019 (COVID‐19), a disease caused by the severe acute respiratory syndrome coronavirus 2 (SARS‐CoV‐2), and first verified in December 2019 in Wuhan, China, has quickly spread throughout the world,^1^ and has since been declared a pandemic by the World Health Organization. Currently, the known routes of SARS‐CoV‐2 transmission are contact, droplet, and airborne routes.^2^, ^3^ Endoscopies, especially upper gastrointestinal endoscopies, are recognized as particularly high risk due to significant aerosol generation.^2^, ^4^ Therefore, the Japan Gastroenterological Endoscopy Society recently issued the “Recommendations for Gastrointestinal Endoscopy in Patients with Novel Coronavirus Infection (COVID‐19),” now widely adopted by endoscopists in Japan. Other countries have also proposed measures, such as the modification of personal protective equipment (PPE), based on the assessment of the patient's infection status.^5^ Globally, endoscopy units are currently faced with the challenge of providing medical care while taking precautions against the spread of infection. One case of an emergency endoscopy performed on a patient with COVID‐19 with a high risk of transmission was reported outside of Japan.^6^ However, in Japan, while there have been instances such as the discovery of an asymptomatic patient's COVID‐19 positive status after endoscopy, there have been no reports of an upper gastrointestinal endoscopy performed on a patient confirmed positive for COVID‐19 thus far. Here, we present the first case report of an emergency endoscopy performed safely on a patient with COVID‐19 for suspected hemorrhagic shock in Japan.

## CASE REPORT

**FIGURE 1:**
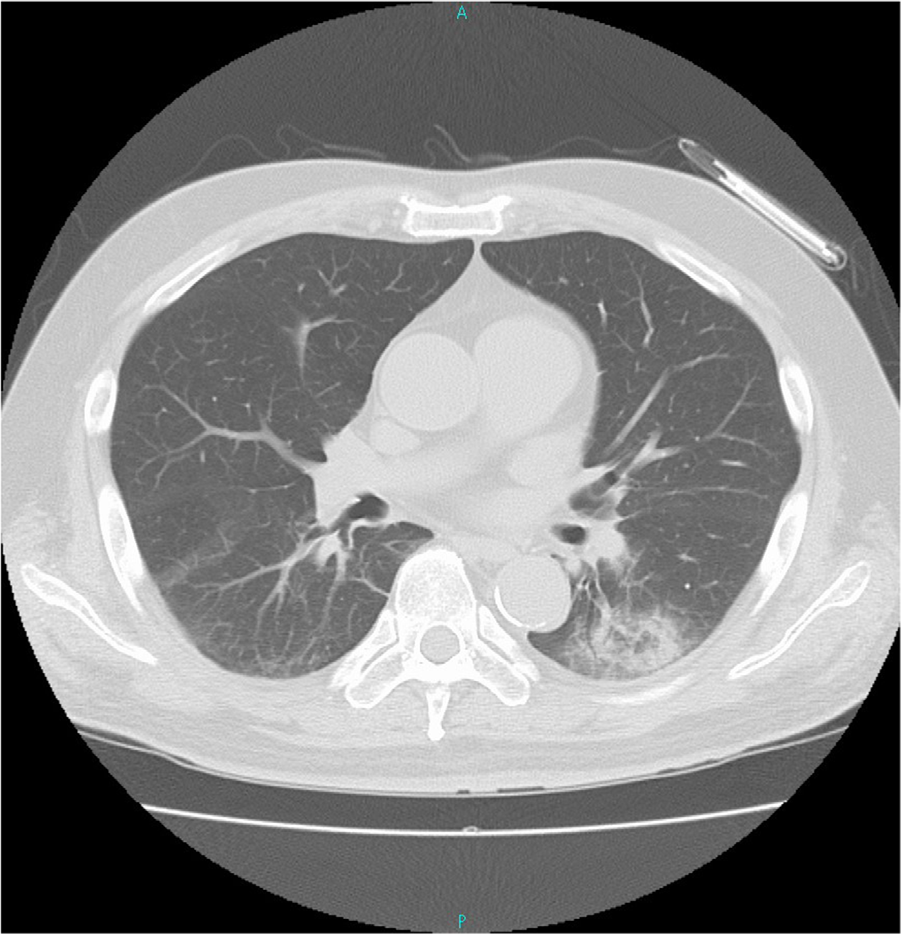
Infiltrative shadows at the center of the inferior lobe of the left lung

**FIGURE 2:**
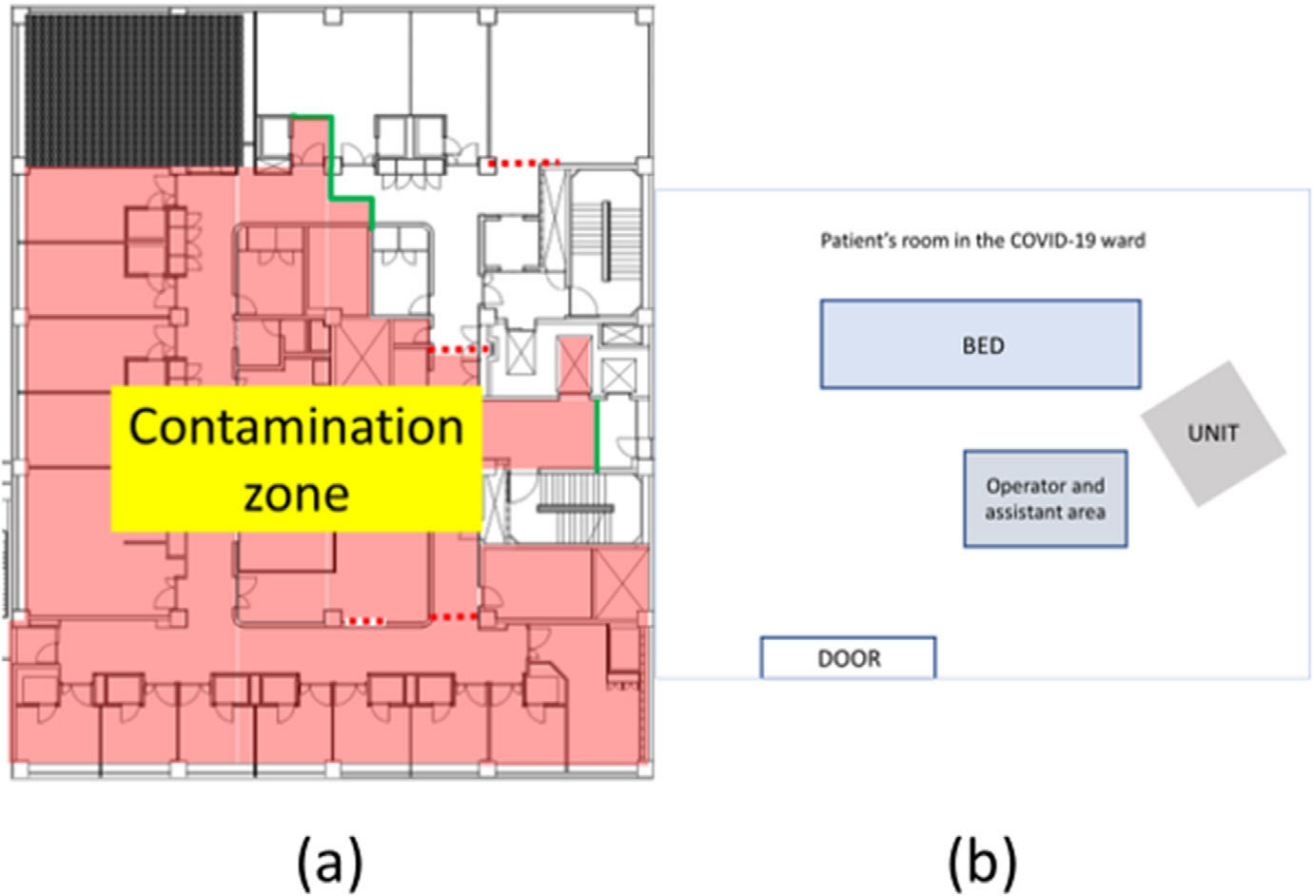
(a) Strict implementation of zoning. (b) The layout of the patient's room at the time of examination

**FIGURE 3:**
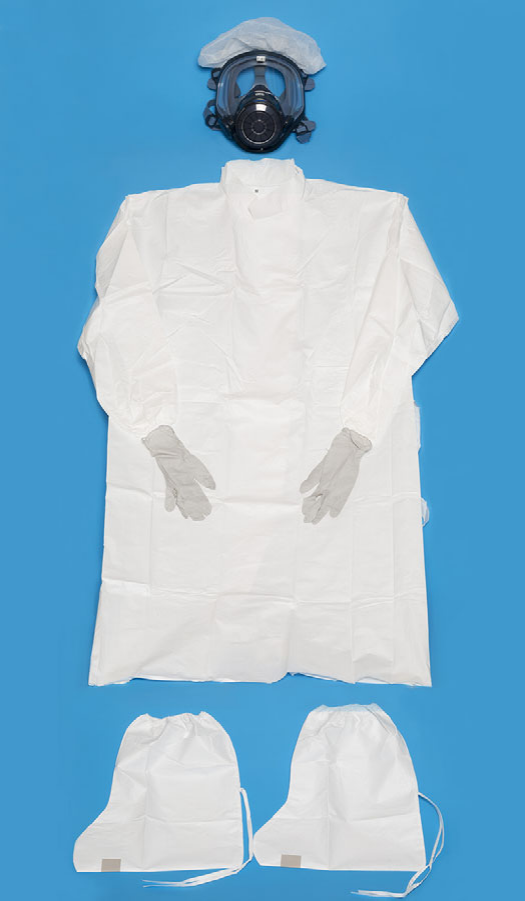
Wearing full personal protective equipment (respirator, double gloves, hairnet, water disposable gown, shoe covers, and face shield/goggles) recommended by various academic societies

**FIGURE 4:**
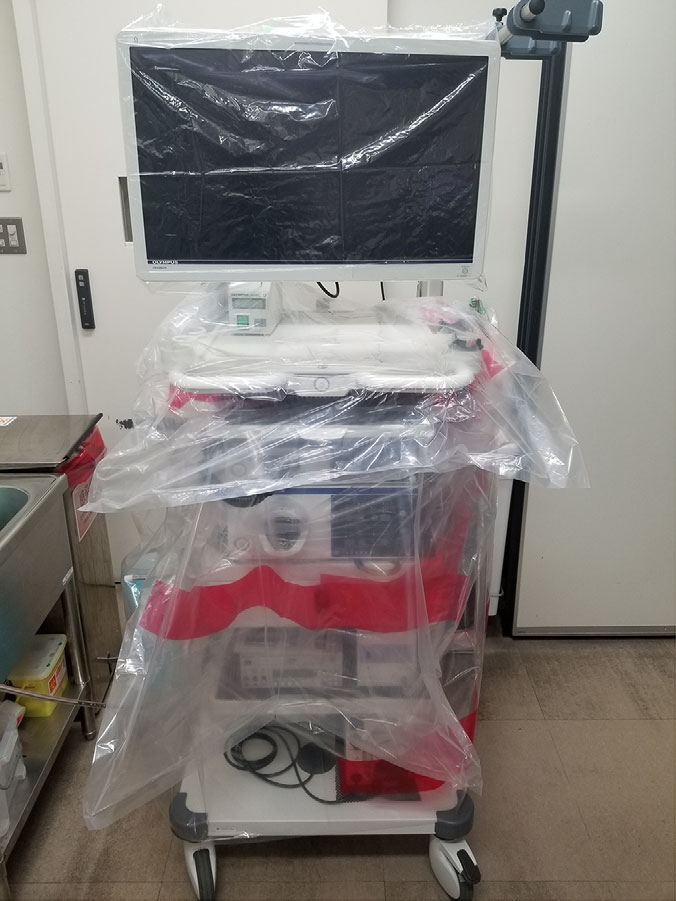
Light source covered in plastic before use

A 65‐year‐old man with diabetic nephropathy and on triweekly maintenance dialysis presented to our hospital with complaints of fever and cough. Oropharyngeal swabs collected on the same day tested positive by the reverse transcriptase‐polymerase chain reaction (RT‐PCR), while computed tomography of the chest revealed infiltrative shadows in the inferior lobe of the left lung (Figure 1). COVID‐19 was thus diagnosed, and the patient was admitted to a specialized COVID‐19 ward where a team of specialists commenced treatment. All rooms in the specialized COVID‐19 ward were private rooms with constant pressure control. Zoning was also strictly enforced for control of infection transmission (Figure 2a). Treatment for COVID‐19 was provided according to Japan's guidelines for novel coronavirus infection and composed of favipiravir, which has also been reported to be effective for COVID‐19 in China for 14 days from the day of admission.^7^ After administration, we observed no marked change in the patient's subjective symptoms or vital signs, including his respiratory status. However, on day 21 of admission, we observed a sudden decrease in blood pressure, a large volume of blackish feces, and progression of anemia. We had avoided an endoscopy until then, but the patient's condition suggested possible hemorrhagic shock due to upper gastrointestinal bleeding. Thus, after deliberation, an emergency endoscopy was performed in the patient's hospital room (located in the contamination zone of a specialized COVID‐19 ward) after informed consent was obtained. We estimated that the endoscopy would pose an extremely high risk of transmission to the medical staff and undertook the procedure in full PPE including a respirator, double gloves, hairnet, water disposable gown, and shoe covers as recommended by various academic societies (Asian‐Pacific Society for Digestive Endoscopy, World Endoscopy Organization, American Society for Gastrointestinal Endoscopy, British Society of Gastroenterology, and others).^2^ In addition, we employed closer‐fitting protective masks with integrated respirators and face shields (Figure 3). The endoscope‐related components used, including the light source and scope, were taken from an endoscopy room where routine examinations were performed, although the light source was covered entirely in plastic before introducing it to the COVID‐19 ward to minimize exposure to aerosols (Figure 4). Two experienced physicians, who were certified endoscopy specialists, and one physician from the special COVID‐19 treatment team were selected to perform the examination. An oral endoscope was chosen for the examination because procedural tools would be required in the case of active bleeding. We performed the examination while the patient was awake because shock vital signs were observed. No barriers were used on the patient side during the examination. Recently, several ideas for improving barriers to be placed on the patient side have been reported.^8^, ^9^ However, no reports on such ideas were available when we performed the endoscopy. The examination was performed in the patient's room with the layout shown in Figure 2b. The examination was completed approximately 3 min after no active bleeding was found from the oral cavity to the horizontal part of the duodenum. Zoning was also strictly enforced after completing the examination, and the PPE were removed with the medical staff observing each other to ensure that no unclean surfaces touched clean surfaces. Regarding the endoscope‐related components used in the examination, small components and plastics were destroyed in the contamination zone, while the scope and light source were wiped thoroughly with 80% ethanol wipes followed by 0.5% accelerated hydrogen peroxide solution wipes. The light source was then transferred to the routine examination room; the scope was sealed in a bag, taken to the cleaning room, washed as usual (according to the ‘WGO Practice Guideline—Endoscope Disinfection’ issued by the World Gastroenterology Organization) by staff wearing full PPE, and then sterilized using 0.3% peracetic acid. The physicians who performed the examination and staff who washed the scope were considered as noncontacts because they were wearing full PPE. They started working as usual from the next day, and they measured and reported their temperature and conditions strictly for 2 weeks. The patient's vital signs subsequently improved with fluid replacement therapy alone. Although the possibility of small intestinal hemorrhage could not be ruled out, due to the anticipated length of time needed to perform an enteroscopy, the associated risk of transmission was deemed too high even with proper measures in place; hence, we decided to monitor the patient's progress. In addition, after the examination, aspirin and heparin calcium, which had been administered before examination, were discontinued, and the dose of vonoprazan fumarate, which had also been administered before examination, was increased from 10 to 20 mg. It was fortunate that following the examination, no findings were observed that would otherwise suggest gastrointestinal bleeding. On day 5 after examination, meals were resumed. A PCR test on day 38 was negative, but a PCR test on day 39 returned a positive result. While another PCR test on day 41 also returned a positive result, based on the high threshold value of this test, we decided that the quantity of RNA was small and posed a minimal risk of transmission; the patient was discharged on day 43. In addition, we closely monitored the health status of all attending medical staff as stipulated by various academic societies and noted no symptoms suggestive of COVID‐19.

## DISCUSSION

Amid the COVID‐19 pandemic, upper gastrointestinal endoscopies in patients with COVID‐19 should be postponed or avoided whenever possible because of the risk of transmission to medical staff.^2^ However, this does not apply to cases deemed highly urgent, such as the present case. To our knowledge, this is the first report to describe an endoscopic examination of a patient positive for COVID‐19 in Japan. Various academic societies have suggested that endoscopies be performed in patients with COVID‐19 in a negative‐pressure room,^2^ but others note that in practice, negative‐pressure rooms are not available to most medical facilities.^5^ Herein, we performed an emergency upper gastrointestinal endoscopy on a patient with COVID‐19 after taking utmost care to reduce the risk of transmission to medical staff. This was done by performing the procedure in an appropriate environment consisting of a specialized COVID‐19 ward (though not under negative‐pressure, but with strict zoning in place), properly assessing the transmission risk posed by the patient, and selecting appropriate PPE. We also took proper precautions to prevent transmission of the virus via used equipment by transporting the used endoscope in a sealed bag, wearing full PPE during the cleaning, and wiping components with 0.5% accelerated hydrogen peroxide solution—all of which likely prevented any subsequent transmission to medical staff. Recently, studies have published ideas for mitigating infection risks, such as using an endoscopy mouthpiece and a respiratory mask and installing a vinyl sheet between a patient and a health professional while also using a mouthpiece with a drape.^8^, ^9^ However, no reports on such ideas were available when we performed the examination; had we implemented them when we treated the present case, we would have been able to further reduce infection risks among the health professionals.

In conclusion, emergency endoscopies can be safely performed on patients with COVID‐19 in a suitable environment by using appropriate PPE and with proper handling of equipment.

## ETHICAL STATEMENT

All procedures followed have been performed in accordance with the ethical standards laid down in the 1964 Declaration of Helsinki and its later amendments.

## CONFLICT OF INTEREST

The authors declare that they have no conflict of interest.

## FUNDING INFORMATION

None.
